# Expanding Culturomics from Gut to Extreme Environmental Settings

**DOI:** 10.1128/msystems.00848-21

**Published:** 2021-08-31

**Authors:** Utkarsh Sood, Roshan Kumar, Princy Hira

**Affiliations:** a The Energy and Resources Institute, New Delhi, India; b Post-Graduate Department of Zoology, Magadh University, Bodh Gaya, Bihar, India; c Department of Zoology, Maitreyi College, University of Delhi, New Delhi, India

**Keywords:** metagenomics, culturomics, extremophiles, microbial dark matter, extremozymes

## Abstract

With the advent of metagenomics, a quest began to identify the dynamics of the microbial communities in different ecological niches. Altogether, this has resulted in identification of microorganisms but is limited to only a small number of phylogenetic groups that can be easily cultured. The majority of metagenomic sequencing data remains unassigned to any known microbial group and is regarded as the “microbial dark matter.” Our group is now working on integrating culturomics (isolation of pure cultures) and metagenomics from extreme environments, particularly from hot water springs and chemically contaminated soils. Our target is to culture the rare extremophiles with biotechnological significance by designing culture media based on inputs from metagenomics. While culturomics integrated with metagenomics has been extensively employed for updating the microbial catalog from the human gut, there is a need to extend this approach to extreme environmental settings to explore the microbial dark matter.

## COMMENTARY

Microbes dominate our planet, as they are found in niches that seem uninhabitable by other living organisms. Although they can easily be visualized using microscopic techniques, historically there is a “1% culturability paradigm” that exists even today (1). Many of these culturable microbes are being used as an important source of useful metabolites and products (2–4). The study of the microbial communities by analyzing the collection of genetic material from different environmental settings, known as metagenomics, revealed that the major fraction of reads remain unassigned and only a small fraction of the data gets assigned to known phylogenetic groups. Thus, the majority of the data sets that remain unassigned represent the “microbial dark matter” (5). Given the importance of pure cultures and the identification of new phylogenetic groups, a quest has begun to identify gut microbial inhabitants using culturomics (6). Somewhere down the line, the era of culturomics has evolved from metagenomics. Culturomics is a high-throughput culture technique, which is an assemblage of different selective/enriched culture conditions coupled with identification techniques such as matrix-assisted laser desorption ionization–time of flight mass spectrometry (MALDI-TOF MS) and 16S rRNA gene sequencing (7). The field of culturomics emerged to procure the “difficult to culture” microbes and, due to the recent advancements in technology, there is a spur in the recovery of low abundance and/or new lineages of microbes. These efforts led to the recovery of a high proportion of microbes from different biomes which were thought to be uninhabitable by microorganisms (8). Attempts are being made to identify the microbial diversity that constitutes the healthy microbiome in the gut of various animals (9, 10). Many diseases have been linked with the dysbiosis of these microbes and restoring their population is now being used as a therapeutic approach (11, 12). Probiotics have also been developed by incorporating the knowledge gained from the metagenomics sequences and culturomics for the identification and culturing of isolates with potential health benefits (13). As discussed, much work has been done on gut microbiota for health and economic benefits, but the unknown “microbial dark matter” from extreme environments is also equally important. The rare extremophiles inhabiting harsh environments have a genetic repertoire that helps them to sustain the harsh environments and, thus, have the potential to act as a source of various extremozymes having industrial importance (14). While culturomics integrated with metagenomics has been extensively employed for updating the microbial catalog from the human gut, the current requirement is to extend this approach to extreme environmental settings to explore the so-called “microbial dark matter.” This commentary highlights the advancements and importance of integrating culturomics with metagenomics for deciphering microbial dark matter in extreme environmental samples.

## ROLE OF MICROBES IN EXTREME ENVIRONMENTS: APPLYING CULTUROMICS TO EXTREME ENVIRONMENTS

In extreme environments, microbes deal with harsh environmental conditions having atypical pH, radiation, temperature, humidity, salt concentrations, and pressure, etc. These microbes are termed extremophiles and play a crucial role in the cycling of nutrients and constitute a reservoir of biocatalysts for future technologies.

These extremophiles utilize key enzymes that can have biotechnological and industrial applications (14). This is because they have adapted to inhabit harsh environments by modulating their protein expressions or by incorporating novel enzymes in their genomes, as required for survival in harsh environments (15). The information gathered from genomes of bacteria has been shown to identify important genes for survival in a particular environment, i.e., the habitat-specific genes (14, 16, 17). The metagenomic data from different environmental samples can be of prime importance in association with culturomics for identifying unknown taxa (18). During the implementation of this technique in an extreme environment, we observed that the culture medium conditions determine the culturable outcomes irrespective of their abundance in the environmental samples. The inputs in terms of genomic information retrieved from metagenome and single-cell genomics, known as metagenome-assembled genomes (MAGs) and single amplified genomes (SAGs), respectively, are now being used to design the culture media based on their functional repertoire (19, 20). These assembled genomes are being used to infer the metabolic pathways for the carbon and nitrogen cycle, the presence of important genes for growth in a particular habitat, the degradation potential, and genes of biotechnological importance (21–23). Further, the extension of metabolic modeling to metagenome-assisted culturomics will be valuable for deciphering the unknown microbial community. Genome-based metabolic models developed for pure cultures are valuable for predicting and cataloging gene functions, metabolic pathways, and reactions. These models (e.g., SEED) are important, as they predict which pathways are utilized by each organism and are not based solely on the presence or absence of genes, and hence play an important role in direct culture conditions (24). Thus, genomic bins and metabolic modeling together can help in accurate predictions of the metabolic potential of environmental communities that can be used as an important input for the cultivation of the unculturable. We believe this can set up a standard condition to obtain the culture of the genomic bins and utilize the information from MAGs for designing the media.

Another important approach is coculturing different microbes depending upon their genomic information. The smaller genomes of many uncultivable organisms recovered from MAGs and SAGs are shown to be deficient for encoding complete pathways for the synthesis of essential nucleic acids and amino acids, and therefore require other microbes to survive in a particular environment (25).

## CHALLENGES AND FUTURE PROSPECTS

Although integrating metagenomics with culturomics seems quite promising, as sequencing data are increasing day by day and hundreds of MAGs are submitted to public databases from a single environment, there are some challenges as well. Most of the predicted genes (around 50%) are not annotated with known functions and therefore information from these genes cannot be used to predict metabolic function. Due to the presence of incomplete pathways and partial knowledge about the function of the majority of the proteins from MAGs, culturing remains a big problem, and the role of syntropic microbes becomes essential. Metagenomic sequencing alone cannot solve these problems and inputs from meta-transcriptomics, meta-proteomics, and metabolomics from environmental samples supplemented with culturing focused on metabolic characterization should also be incorporated (22).

Progress in the field of computational biology is also very important. As most of the proteins from these MAGs are hypothetical, the development of newer bioinformatic tools to analyze and annotate the exponentially growing hypothetical proteins discovered from metagenomics is of great importance. These genes are key for identifying novel pathways related to microbial metabolism, newer links in biogeochemical cycles, and also for identifying the role of community dynamics within a niche. The genetic information is imperative for determining specific electron donors, carbon sources, and electron acceptors, and for selecting the pathways that have to be supplemented by the culture medium. The culturing medium is to be designed based on the genomic information that can assist in isolating microorganisms with reduced genomes. Meta-omics can also be useful in certain cases where genomic information is inadequate, for determining the active bacteria in selected conditions (23). The information from the metaomics reveals the active metabolic functions and provides critical inputs regarding the substrates that are preferably catabolized by organisms, particularly extremophiles inhabiting a particular niche with atypical conditions. We are sure that with advancements in meta-omics and culturomics, newer microbes and their role in maintaining the ecosystem at a particular habitat will be identified. The newer information gathered will substantially influence the existing knowledge for culturing and the biotechnological potential of the extremophiles.

Further, to date, no specific guidelines or system exists for the nomenclature of unculturable microbes. There is also an urgent need to develop a nomenclature scheme for the microbes whose existence is deciphered using metagenomic information. Nowadays, several journals are publishing novel species but without following the guidelines of novel species characterization, thus stringent steps should be taken to characterize these isolates and a microbial consortium should be established to curate the novel isolates and to test them based on taxonomy guidelines, irrespective of their publication in any journal.

**FIG 1:**
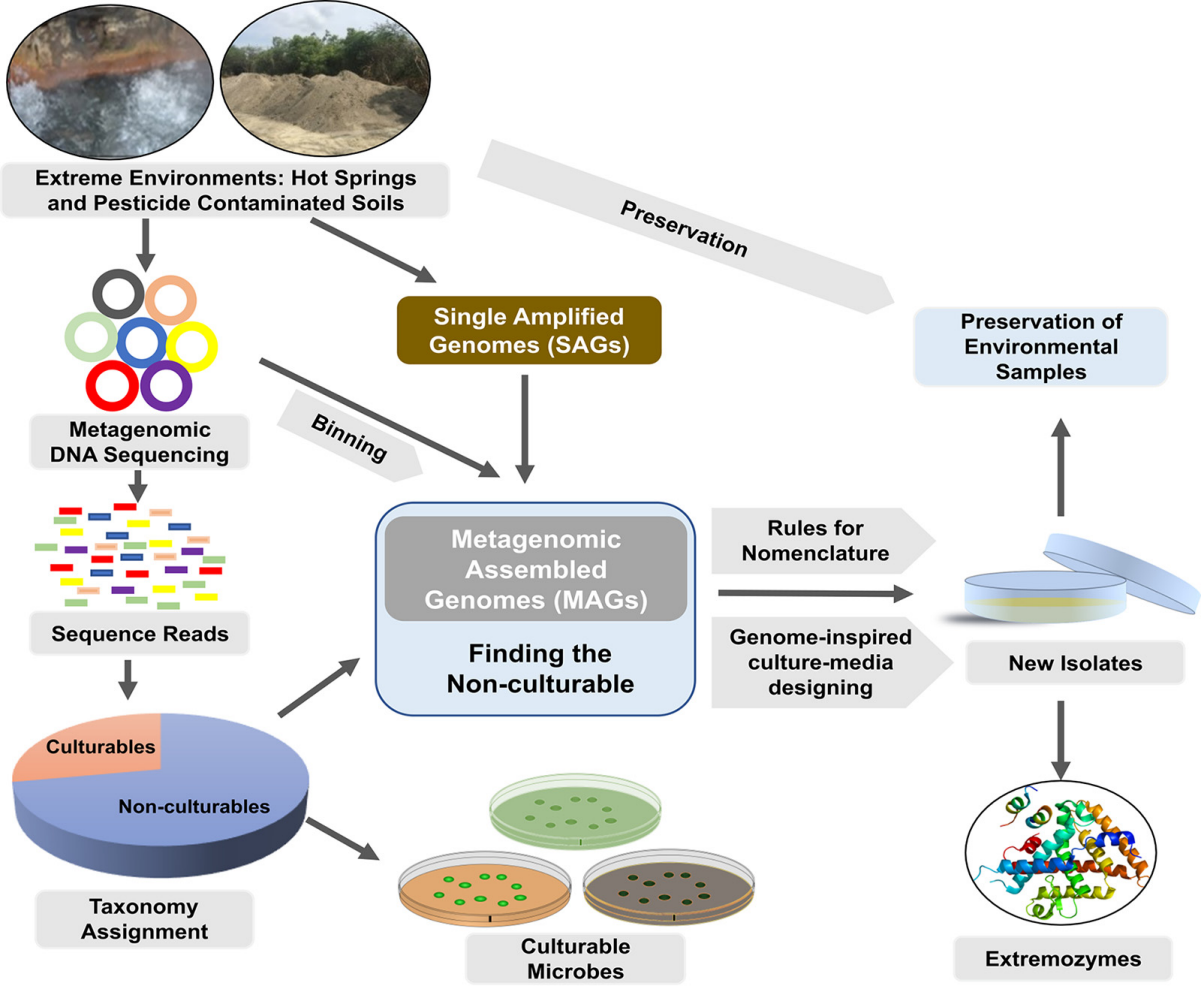
Schematic representation of the workflow for designing media based on genomic information from metagenomic assembled genomes (MAGs). New isolates can act as a source of novel extremozymes. Preservation of environmental metagenomes and newer isolates is also important and can act as a future resource for environmental microbiology.

Additionally, there is a need to conserve environmental samples from important sites as pure cultures, as they are a valuable resource for environmental microbiology and can also be used to study the temporal changes of microbes at that particular site. Our group is working on two extreme environments, *viz.* hot water springs in the Himalayan regions and a set of pesticide-contaminated sites. We have gathered multiomics data that are now being implemented to attempt culturing of the as-yet-unknown microbes from these two environments. By integrating this knowledge, we conclude that culturing the organisms is important for understanding them in a particular niche and that genomes play a vital role in understanding how to culture these bacteria. We summarize this commentary with a projected workflow that we are adopting to culture extremophiles with industrially important extremozymes and that can be further applied to other environmental samples (Fig. 1).
